# Supplementary material for: SARS-CoV-2 Transmission in Belgian French-Speaking Primary Schools: An Epidemiological Pilot Study
Source: Viruses. 2022 Oct 6;14(10):2199. doi: 10.3390/v14102199 (PMC9612207; doi:10.3390/v14102199)
Supplement: Supplementary file 1 [file viruses-14-02199-s001.zip › viruses-1920498-supplementary.pdf]

Supplementary data:

Table S1: Weekly reporting of COVID-19 cases by the school health promotion department (SHPD).

School n°	Week		Reason for testing	Type of contact	School n°	Week		Reason for testing	Type of contact
School n°1	W7	1 pupil	Symptoms	Index	School n°4	W10	1 pupil	Symptoms	Index
	W11	1 SS	Unknown			W11	2 pupils	Family contact	Index
	W12	1 pupil	Symptoms	Index		W17	1 pupil	Family contact	Index
School n°2	W9	1 pupil	Family contact	Index	School n°5	W3	1 pupil	Symptoms	Index
	W11	3 SS	Symptoms	Index		W3	1 pupil	Symptoms	Secondary
	W11	6 pupils	Contacts	Secondary		W4	1 SS	Other	Index
	W11	1 pupil	Symptoms	Index		W4	1 pupil	Symptoms	Index
	W11	1 pupil	Family contact	Index		W4	1 pupil	Family contact	Index
	W12	6 pupils	Pupil contact	Secondary	School n°7	W6	2 pupils	Symptoms	Index
	W12	2 pupils	Family contact	Index		W8	1 pupil	External contact	Index
	W12	2 pupils	Symptoms	Index		W9	1 pupil	Family contact	Index
	W12	5 pupils	Contact	Secondary		W10	2 pupils	Family contact	Index
	W14	2 pupils	Family contact	Index		W11	2SS	Symptoms	Index
	W16	1 SS	Symptoms	Index	School n°8	W9	2 pupils	Family contact	Index
	W17	2 pupils	Family contact	Index		W9	SS	Other	Index
	W17	1 pupil	Symptoms	Index		W10	1 pupil	Symptoms	Index
	W17	1 pupil	Pupil contact	Secondary		W12	1 SS	Unknown	Index
	W18	1 pupil	Family contact	Index		W13	1SS	Symptoms	Index
School n°3	W17	1 pupil	Family contact	Index					

Weekly reported numbers of pupils and staff with confirmed SARS-CoV-2 infections by SHPD, reason for testing (symptoms or high risk contact) and suspected source of infection (index case: contamination outside the school environment or secondary case: probable infection in the school environment). The weeks mentioned correspond to the calendar weeks of the year 2021.

Table S1: Weekly reporting of COVID-19 cases by the school health promotion department (SHPD) (continued).

School n°	Week		Reason for testing	Type of contact
School n°9	W8	1 pupil	Family contact	Index
	W8	1 pupil	Pupils contact	Secondary
	W9	1 pupil	Family contact	Index
	W10	1 pupil	Pupil contact	Secondary
	W12	1 pupil	External contact	Index
	W22	1 pupil	Symptoms	Index
School10	W12	SS	Symptoms	Index
	W17	SS	Symptoms	Index
	W18	1 pupil	Family contact	Index
	W20	1 pupil	Symptoms	Index
	W20	1 pupil	External contact	Index
School 11	W6	1 pupil	Other	Index
	W17	1 pupil	Family contact	Index
	W18	1 pupil	Family contact	Index
	W19	1 pupil	General testing	Secondary

Weekly reported numbers of pupils and staff with confirmed SARS-CoV-2 infections by SHPD reason for testing (symptoms or high risk contact) and suspected source of infection (index case: contamination outside the school environment or secondary case: probable infection in the school environment). The weeks mentioned correspond to the calendar weeks of the year 2021.

Detailed description of SARS-CoV-2 whole-genome sequences method.

Positive samples presenting Ct values < 25 were then sequenced starting from the saliva samples stored at −80°C until sequencing. Two hundred µl of the transport media were used

for total nucleic acid extraction using the MagMAX™ Viral/Pathogen II Nucleic Acid Isolation Kit according to the manufacturer instructions (Cat. No. A48383, ThermoFisher Scientific). The amplicon-based Illumina COVIDSeq protocol (Illumina Inc, USA) in combination with the ARTIC v4 primers pools (https://artic.network/) was used for sequencing according to manufacturer's instructions. Briefly, a first strand cDNA was generated and amplified using a multiplex polymerase chain reaction (PCR) protocol, producing amplicons across the whole SARS-CoV-2 genome. The amplicons were later processed for tagmentation and adapter ligation using IDT for Illumina Nextera UD Indexes (Illumina Inc, USA). After additional PCR amplification of the tagmented amplicons and a bead-based cleanup, libraries were pooled together in a single tube. The final pool was quantified using Qubit 2.0 fluorometer (Invitrogen Inc.) and fragment size was evaluated using an Agilent 2100 Bioanalyzer (Agilent Inc). The pooled library was diluted to a final concentration of 100pM for a single read (1 x 150bp) sequencing on a NextSeq 1000 instrument. Generated fastq files were uploaded on the cloud-based ASP-IDNS®−5 analysis software (SmartGene). Analysis was made using the "SARS-CoV-2 full genome" pipeline version 2.5.0_COV_v0.2. Briefly, reads were automatically filtered for low quality sections. The resulting reads were mapped against the SARS-CoV-2 (Wuhan-hu-1/2019 (MN908947)) reference genome and mutations were detected in a quantitative manner (% reads aligned). A consensus genome was generated using a 40% cut-off for base determination and a minimal number of 30 reads per position. Online Nextclade version 2.3.0 software was used as a first sequence aligner, allowing comparison to the Wuhan-hu-1/2019 (MN908947) SARS-CoV-2 reference genome and permitting a clade assignment (https://clades.nextstrain.org). FASTA sequences were also submitted to the Pangolin (4.1.1) COVID-19 Lineage Assigner.
